# Strong tuberculin response after BCG vaccination is associated with low multiple sclerosis risk: a population-based cohort study

**DOI:** 10.1093/ije/dyac039

**Published:** 2022-03-12

**Authors:** Ola Nakken, Trygve Holmøy, Hein Stigum, Kjell-Morten Myhr, Jesper Dahl, Einar Heldal, Haakon E Meyer

**Affiliations:** Department of Neurology, Akershus University Hospital, Lørenskog, Norway; Department of Neurology, Akershus University Hospital, Lørenskog, Norway; Institute of Clinical Medicine, University of Oslo, Oslo, Norway; Department of Physical Health and Ageing, Norwegian Institute of Public Health, Oslo, Norway; Department of Community Medicine and Global Health, University of Oslo, Oslo, Norway; Department of Clinical Medicine, University of Bergen, Bergen, Norway; Neuro-SysMed, Department of Neurology, Haukeland University Hospital, Bergen, Norway; Department of Physical Health and Ageing, Norwegian Institute of Public Health, Oslo, Norway; Department of Physical Health and Ageing, Norwegian Institute of Public Health, Oslo, Norway; Department of Physical Health and Ageing, Norwegian Institute of Public Health, Oslo, Norway; Department of Community Medicine and Global Health, University of Oslo, Oslo, Norway

**Keywords:** Neurology, multiple sclerosis, neuroinflammation, neuroepidemiology, immune responses, Bacillus Calmette–Guérin, cohort study

## Abstract

**Background:**

Multiple sclerosis (MS) is characterized by inflammatory lesions in the central nervous system involving pro-inflammatory T-cells. Immune dysregulation is well described in prevalent disease, but it is not known whether this precedes disease development. Bacillus Calmette–Guérin (BCG) vaccination ameliorates MS-like disease in mice. In people vaccinated with BCG, the tuberculin skin test (TST) offers a standardized measure of a T-cell-mediated immune response. We therefore hypothesized that the strength of the TST response after BCG vaccination is associated with subsequent MS risk.

**Methods:**

Using data from a Norwegian tuberculosis screening programme (1963–1975), we designed a population-based cohort study and related the size of TST reactions in individuals previously vaccinated with BCG to later MS disease identified through the Norwegian MS registry. We fitted Cox proportional hazard models and flexible parametric survival models to investigate the association between TST reactivity, MS risk and its temporal relationship.

**Results:**

Among 279 891 participants (52% females), 679 (69% females) later developed MS. Larger TST reactivity was associated with decreased MS risk. The hazard ratio for MS per every 4-mm increase in skin induration size was 0.86 (95% confidence interval 0.76–0.96) and similar between sexes. The strength of the association persisted for >30 years after the TST.

**Conclusion:**

A strong *in vivo* vaccine response to BCG is associated with reduced MS risk >30 years later. The immunological mechanisms determining TST reactivity suggest that skewed T-cell-mediated immunity precedes MS onset by many decades.

Key Messages

- Tuberculin skin test (TST) responses after Bacillus Calmette–Guérin (BCG) vaccination are dependent on T-cell-mediated memory recall immunity, involving key immune elements also operating in multiple sclerosis (MS).
- It is not known whether immune dysregulation precedes MS disease development.
- We found that a strong tuberculin response after BCG vaccination was associated with lower MS risk and that the association persisted for >30 years after the TST.
- The immunological mechanisms determining TST reactivity suggest that skewed T-cell-mediated immunity precedes MS onset by many decades.

## Introduction

The prevalence of multiple sclerosis (MS) is increasing and a total of 2.8 million people live with MS worldwide.^1^ MS is driven by inflammation in the central nervous system involving both T-cells, B-cells and myeloid cells.^2^ The prevailing genetic association with human leukocyte antigen (HLA) class II and other immune-related genes,^3^ as well as indirect evidence from animal models, suggest that pro-inflammatory CD4 T-cells play a primary role in disease initiation. Although no target antigen has been firmly established, the clonal expansion of B-cells in the brain, cerebrospinal fluid and cervical lymph nodes are characteristic of an antigen-driven adaptive immune response.^4^

The main environmental risk factors associated with MS, including Epstein–Barr virus (EBV) infection,^5^ smoking,^6^ low vitamin D levels^7^ and adiposity in early life,^8^ may facilitate pro-inflammatory adaptive immune responses. Whereas several studies suggest defective peripheral B- and T-cell immune tolerance in people with MS,^9^ as for other autoimmune diseases, it is not known whether, and if so when, immune dysregulation precedes disease development. To address this question, we have used population-based data from the compulsory Norwegian tuberculosis screening programme, including a quantitative measure of a post-vaccination immune response against Bacillus Calmette–Guérin (BCG).

BCG is an attenuated strain of *Mycobacterium bovis* used extensively since 1921. BCG elicits strong T-cell-mediated immune responses, involving both pro-inflammatory and regulatory T-cells,^10^ as well as long-term epigenetic changes of innate immune cells—so-called trained immunity.^11^ Thus, both adaptive and innate immunity may contribute to the pleiotropic effects of BCG.^12^ In addition to partial protection against tuberculosis and treatment for bladder cancer, BCG ameliorates experimental autoimmune encephalomyelitis ^13^ through suppression of encephalitogenic Th17-cells in the central nervous system.^13^ Moreover, BCG vaccination has been shown to prevent inflammatory disease activity in patients with early stages of MS.^14^ Immune responses induced or regulated by BCG may therefore be relevant in MS.

In the early twentieth century, Norway had one of Europe’s highest tuberculosis rates.^15^ In 1947, responding to this threat and motivated by new possibilities for case finding and treatment, the Norwegian Parliament passed an act making participation in a national tuberculosis control programme compulsory for all >14 years of age. The programme included tuberculosis testing through chest X-ray, tuberculin skin test (TST) and height and weight measurement. All individuals with a negative TST and no signs of tuberculosis disease were strongly encouraged to accept vaccination with BCG.^16^^,^^17^

The TST has been employed since the early twentieth century primarily to detect infection with tuberculosis^18^ but also to document the immunological response to BCG vaccine. In Norway, even those who had a previously documented BCG vaccination, commonly as part of the national school vaccination programme at age 12–14 years, underwent testing with the TST in the compulsory screening programme. The test represents a classical delayed-type hypersensitivity response to the intradermal application of antigens derived from tubercle bacilli. It produces a skin induration in patients previously sensitized towards the antigen, either from BCG vaccination or from infection with *Mycobacteriumtuberculosis* or other mycobacteria. A positive response expresses an intact T-cell-mediated memory recall immunity^19^ involving many of the key immune mediators operating in MS. Thus, skin biopsies taken at various time points after the TST reveal infiltration and a strong dependence of CD4 T-cells.^20^ Furthermore, Th17 activity has been shown to facilitate a post-BCG vaccination response^21^ and, conversely, lack of Th17 activity inhibits this response.^22^ Th17-cells may also have a pivotal role in MS pathogenesis.^2^^,^^23^

In this study, we identified a large group of young individuals characterized by having undergone a standardized immunization (BCG vaccine) followed by a TST and explored whether those who later developed MS had had a deviating tuberculin response after BCG vaccination in early life.

## Materials and methods

### Study population

During 1948–1975, a compulsory screening programme for tuberculosis in the general population was performed by the National Mass Radiography Service for all individuals aged ≥15 years in 18 of Norway’s 19 counties. The capital of Oslo had a separate screening programme. Non-attendance was mainly due to ‘acceptable excuses’ such as already under control or treatment for tuberculosis, in military service or in hospital. The overall attendance rate among eligible individuals was 80–85%.^24^ Computerized records of examinations conducted between 1963 and 1975 comprise chest X-ray investigations, TST measurements, documentation of BCG vaccination status and measured heights and weights from 1 911 598 individuals. The national school vaccination programme in Norway was gradually implemented from the early 1950s during the sixth to eighth grades (ages 12–14 years) with very high coverage. Hence, in this late stage of the tuberculosis screening programme, a large proportion of young participants were BCG-vaccinated.^17^ At the same time, the incidence of tuberculosis in Norway dropped to a very low rate (<2/100 000) in these age groups.^15^^,^^17^

BCG was produced at the Bergen State BCG Laboratory (Bergen, Norway) using the Swedish Gothenburg strain until 1973^25^ and thereafter by Statens Serum Institute (Copenhagen, Denmark). Liquid BCG was gradually replaced by freeze-dried BCG between 1959 and 1973.^26^

**Figure 1:**
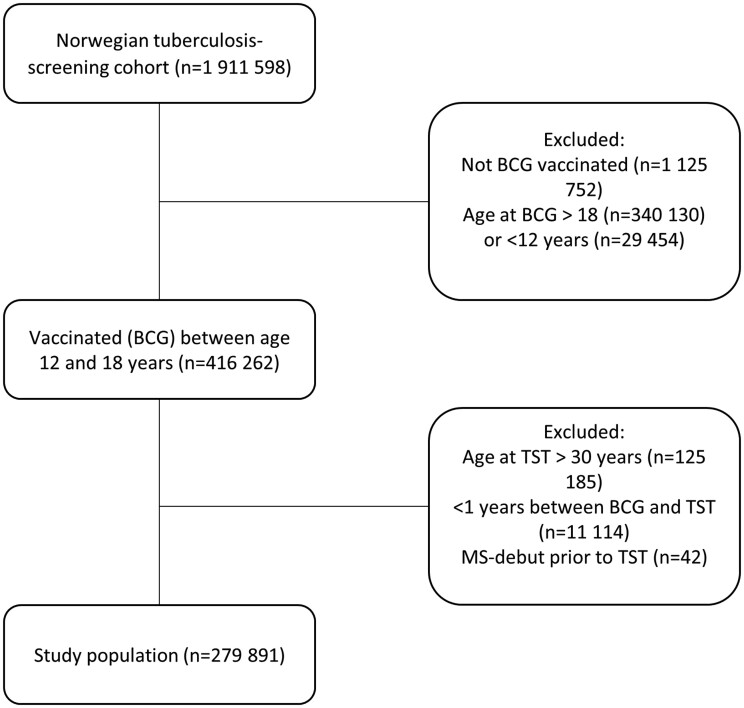
Selection of participants. BCG, Bacillus Calmette–Guérin; TST, tuberculin skin test; MS, multiple sclerosis.

We aimed to include young, vaccinated individuals with a succeeding TST result (Figure 1). In total, 279 891 individuals matched our pre-defined criteria of being aged 12–18 years at BCG vaccination (85% of whom were 12–14 years old) and 13–30 years old at examination with a TST. If a participant was screened more than once, the first TST was selected. Only the calendar year for vaccination and TST were available. Therefore, to ensure that the vaccination had preceded the TST, the sample was limited to individuals with a previously documented BCG vaccination at least a year before examination with a TST. Ultimately, we excluded individuals registered with onset of MS symptoms prior to the TST.

### TST

For the TST, the adrenaline–Pirquet method was used.^27^ A drop of concentrated tuberculin (Old tuberculin) was placed on the volar aspect of the underarm. Adrenaline was added to the solution throughout the period to increase the biological activity. Two 5-mm-long scratches were then produced in the superficial layer of the skin by means of a pen nib and the sites left to dry for 5 min. After 48 h (maximum 72 h), the largest of the two infiltrates was recorded in mm. The tuberculin skin infiltrate (induration) size was measured according to strict national guidelines during the screening programme.^15^ In this paper, ‘TST’ refers to the adrenaline–Pirquet test, whereas we use TST reactivity when referring to the measured TST induration sizes.

We modelled TST reactivity in three ways: (i) continuously (4-mm increments), (ii) by quartiles of distribution and (iii) binary (0–3 or ≥4 mm). The latter was the definition used for positive or negative TST during the Norwegian mandatory mass tuberculosis screening and BCG vaccination programme.^15^

### Case ascertainment and data linkage

MS cases were identified through the Norwegian MS registry and biobank (hereafter referred to as ‘the MS registry’). The MS registry is based on informed consent. Its operative period dates back to 2001. From that time onwards, both incident and prevalent cases have been registered in the database by local neurologists. Date of symptom onset (clinical isolated syndrome (CIS) or a less defined symptom) is recorded based on hospital records or patient recall. Thus, for many cases in the MS registry, the recorded date of symptom onset precedes the date of registry initiation by years and decades. Although it is a nationwide registry, in adjacent years following registry initiation, geographical coverage was variable. The overall completeness of the registry has gradually increased and is currently reported as being close to 80% of that calculated from an administrative health register (Norwegian Patient Registry). The MS registry is nationwide but geographical coverage is nevertheless still somewhat variable.^28^ From the MS registry we collected information on date of symptom onset, date of diagnosis, disease course at diagnosis and county of residence.

We retrieved vital status and emigration from the National Population Registry. Data were extracted on 26 November 2020. Register linkage was facilitated through the unique personal identification number given to all Norwegian citizens.

### Statistical analysis

Each participant contributed the follow-up time from the date of the TST to the date of MS symptom onset, death, emigration or the end of study follow-up (26 November 2020), whichever came first.

Hazard ratios (HRs) and their 95% CIs were calculated using Cox proportional hazard models with time since TST (years) as the time variable. In one basic model, age at BCG and time between BCG and TST were included as the only covariates. These factors are considered the most important determinants of tuberculin reactivity in BCG-vaccinated individuals.^19^^,^^29^ In our main multivariable-adjusted model, we added sex, BMI, birth year and county of residence as covariates.

We used TST reactivity as a continuous exposure. To investigate the dose–response relationship, we fitted our main multivariable Cox model using restricted cubic splines (with three knots) in the continuous TST measurement. We also show results of TSTs categorized into quartiles.

Between-sex heterogeneity was tested using a likelihood ratio test between the main model and a model including an interaction term of TST reactivity and sex. If increasing HR was observed with increasing time between BCG and TST, it could potentially imply effects from infection with *M. tuberculosis* or non-tuberculous mycobacteria.^19^ We therefore also tested the interaction between TST reactivity and time between BCG and TST in a similar manner.

For investigating the potential time-varying effects (non-proportional hazards), we fitted flexible parametric models (specified the same way as the main model above) using the stpm2 package in STATA.^30^ Here, we allowed the effect of 4-mm increments in TST reactivity to vary over the analysis time using a spline. We then plotted predicted HRs using both time since screening and age under study as timescales.

In sensitivity analyses, multivariable Cox models using continuous TST reactivity were performed: (i) limited to individuals residing in counties characterized by above average MS registry coverage; (ii) excluding the first 5 years of follow-up. The latter was done to minimize the possibility of including screening participants in a prodromal phase of MS.

The statistical program STATA (version SE 16.1) was used for calculations.

## Results

The 279 891 participants selected from the tuberculosis screening programme (52% female) collectively contributed to 13 776 184 person-years of observation time and had a mean follow-up of 49.2 years [standard deviation (SD) 7.6]. We identified 679 cases (69% female) in the MS registry with a mean follow-up of 21 years (SD 10.5).

Although ages at BCG were similar, cases were somewhat younger than non-cases at the time of the TST. Consequently, the elapsed time between these events was shorter among cases (Table 1).

**Table 1:** Tuberculosis screening cohort characteristics at time of tuberculin skin test, according to multiple sclerosis status and sex

	Females		Males	
	MS	Non-MS	MS	Non-MS
Participants (*N*)	469	145 559	210	133 653
Age at BCG [mean years (SD)]	13.3 (1.4)	13.4 (1.4)	13.2 (1.3)	13.2 (1.3)
Age at TST [mean years (SD)]	19.7 (4.3)	21.1 (4.8)	18.9 (3.9)	20.7 (4.8)
Years between BCG vaccination and TST [mean (SD)]	6.4 (4.3)	7.8 (4.6)	5.7 (3.9)	7.4 (4.6)
Birth year [mean (SD)]	1949 (4.9)	1947 (5.4)	1950 (4.5)	1948 (5.4)
BMI [mean kg/m^2^ (SD)]	22.0 (3.1)	22.1 (2.8)	22.3 (2.8)	21.9 (2.6)
TST infiltrate size [mean mm (SD)]	5.5 (2.6)	6.0 (2.9)	6.1 (2.5)	6.5 (2.8)

MS, multiple sclerosis; BCG, Bacillus Calmette–Guérin; TST, tuberculin skin test; SD, standard deviation.

**Figure 2:**
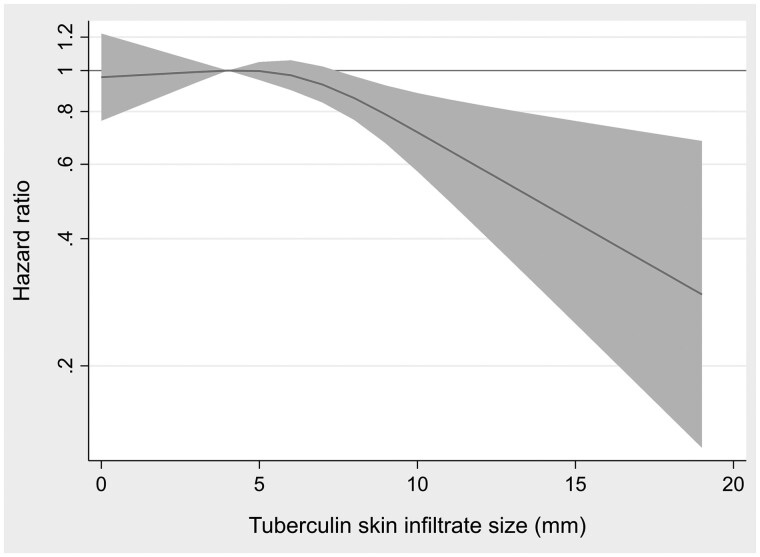
Dose–response: multiple sclerosis hazard ratio by tuberculin skin infiltrate size. Shaded area represents 95% CIs.

In general, larger TST reactivity was associated with decreased MS risk. Entering the TST as a continuous variable, a 4-mm increase was associated with a multivariable-adjusted HR of 0.86 (95% CI 0.76–0.96) (Table 2). The associated risk reduction for MS was mainly driven by those with the largest infiltrate sizes (Table 2 and Figure 2). Using the pre-defined dichotomy, the HR of MS was 0.86 (95% CI 0.71–1.03) in those with a positive (≥4 mm) compared with those with a negative (0–3 mm) TST reaction.

**Table 2:** Risk of multiple sclerosis by categories of tuberculin skin test reactivity

TST category	Participants	Multiple sclerosis	Person-years	Age-adjusted^a^ HR (95% CI)	Multivariable adjusted^b^ HR (95% CI)
HR per 4-mm increment in infiltrate size	279 889	679	13 776 184	0.83 (0.75–0.93)	0.86 (0.76–0.96)
Quantiles of distribution^c^					
First quartile	83 756	233	4 165 549	0.99 (0.81–1.20)	0.95 (0.78–1.15)
Second quartile	67 159	182	3 317 245	Reference	
Third quartile	73 392	178	3 588 191	0.95 (0.78–1.17)	0.95 (0.78–1.17)
Fourth quartile	55 584	86	2 705 200	0.65 (0.50–0.84)	0.65 (0.50–0.85)

a Adjusted for age at TST and age at BCG.

b Multivariate adjusted for sex, age at BCG, age at TST, birth year, county of residence and BMI.

c First quartile: 0–4 mm; second quartile: 5–6 mm; third quartile: 7–8 mm; fourth quartile: 9–50 mm.

TST, tuberculin skin test; BCG, Bacillus Calmette–Guérin; HR, hazard ratio.

**Figure 3:**
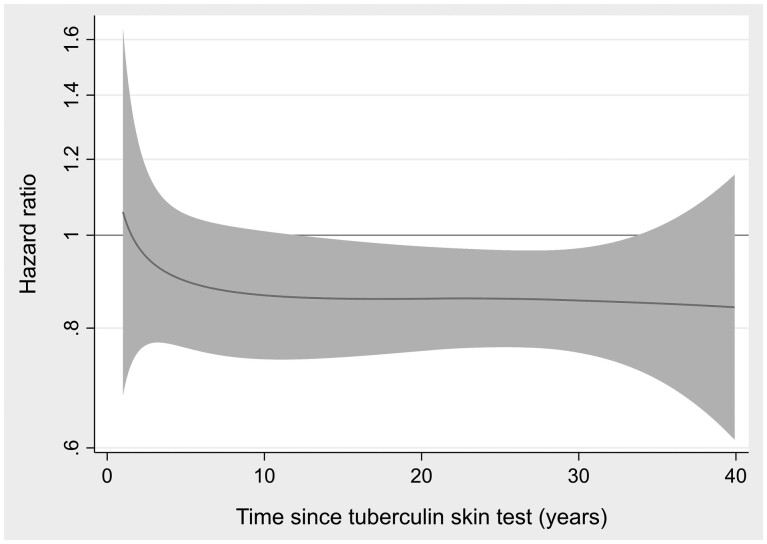
Proportional hazards: multiple sclerosis hazard ratio (for 4-mm increments in tuberculin skin infiltrate size) by time since tuberculin skin test. Shaded area represents 95% CIs.

The association between a 4-mm increase in the TST reactivity and MS risk was stable over the observation time (proportional hazards) and persisted for more than three decades (Figure 3). Changing the timescale to age under study, the strength of the association was also similar for different ages at MS symptom onset (Supplementary Figure S1, available as Supplementary data at *IJE* online).

There was no effect modification by either sex (*P* = 0.99) or elapsed time between BCG and TST (*P* = 0.65).

To address the possibility of reverse causality, we performed a sensitivity analysis excluding the first 5 years of follow-up. Here, 42 cases were excluded leaving 637 cases among 278 719 participants. The results remained similar. The HR for MS was 0.86 (95% CI 0.76–0.96) for each 4-mm increment in TST reactivity. Restricted analysis to participants residing in 10 counties characterized by above average MS registry coverage left 168 842 participants and 451 cases. The association between TST and MS risk persisted [HR for MS was 0.82 (95% CI 0.71–0.94) for each 4-mm increment in TST reactivity].

## Discussion

In this population-based study, we show that a strong post-BCG vaccination immune response, as measured by TST reactivity, is associated with decreased risk of MS >30 years later.

Both BCG vaccination and infection with *M.tuberculosis* and other mycobacteria may contribute to TST results.^31^ All individuals under study had undergone BCG vaccination, typically 5–10 years prior to the TST. In this temporal context, and since the strength of the association was unaffected by the time between BCG and TST,^31^ the variation in the TST reactivity in our study population most probably should reflect individual variation in immune responses to BCG rather than environmental exposure to mycobacteria. Accordantly, this study included young participants screened in the late stage of the screening programme when the incidence of tuberculosis in Norway was low.^15^^,^^17^ Moreover, serological studies from Italy and Japan show increased antibody reactivity against *Mycobacteria avium* subsp. *paratuberculosis* in people with MS,^32^^,^^33^ suggesting that any effect of mycobacteria infection on MS risk would be opposite to our results.

Prospective studies on healthcare use,^34^ cognitive performance^35^ and serum biomarkers of neurodegeneration^36^ suggest a long prodromal phase in MS. Although we cannot exclude that our findings represent reverse causality with disease-induced alterations of immune responses, this seems unlikely since the strength of the association did not vary over the observation time, was present decades before symptom onset and persisted when excluding the first 5 years of follow-up. Rather, the observations are compatible with genetic or acquired variations in immune regulation.

Although no specific shared gene or genes are known, genetic factors linked to both tuberculosis,^37^ TST reactivity^38^^,^^39^ and MS susceptibility^3^ include HLA class II and other genes that influence T-cell-mediated immunity and could potentially explain the observed association. Moreover, environmental factors can affect both MS risk and TST reactivity. Whereas EBV infection is a major environmental risk factor for MS,^5^ psychosocial stress has been associated with both MS risk and with diminished T-cell-mediated immune responses including TST and with EBV reactivation.^40^ Our findings are compatible with restrained T-cell-mediated immunity making some individuals more prone to viral infections and reactivations linked to MS disease initiation.

Early-life obesity is also associated with increased MS risk.^8^ Reports linking nutritional status and body weight to TST reactivity are conflicting, with malnutrition possibly causing TST anergy.^41^^,^^42^ Regardless, BMI was included in our adjusted analyses and should not have biased our results. Both smoking^6^ and low levels of vitamin D,^7^ other potential risk factors for MS, have no known direct effects on delayed-type hypersensitivity reactions. Nevertheless, these factors impact the immune system in a variety of ways^43–45^ and are potential unmeasured confounding factors in our study.

Our study has limitations. Case ascertainment was not complete. If missing data are related to the exposure or confounding factors, bias can be introduced. First, whereas symptom onset in some cases was dated back to a calendar time close to the TST screening, the Norwegian MS registry did not include incident patients before 2001. Hence, the mean age at onset in this case cohort was older (mean 40 years) than is typical for MS (closer to 30 years). Although age at onset may relate to disease phenotype, it is not believed that it directly relates to the underlying pathophysiology of the disease.^46^ In accordance, no clear variance in the strength of the observed association was seen across different ages at onset. Nevertheless, we cannot exclude that a relative under-ascertainment of people diagnosed with MS at young ages has affected our results. Thus, individuals with a strong tuberculin response and a genetic predisposition for MS could get the disease at younger ages than those with a weak tuberculin responses. Second, MS registry coverage varies between counties. Although the distribution of tuberculosis infections and consequently the frequency of mass screening visits varied geographically, we know of no such variation in either the BCG strains used or the strict protocols for TST reading. Our sensitivity analysis restricted to participants residing in areas with above average MS registry coverage produced stronger effect estimates. This further supports our assumption of no significant bias from incomplete case finding.

In conclusion, we have demonstrated that strong BCG vaccine responses are associated with low MS risk more than three decades later at a population level. Vaccine responses represent an opportune way to investigate whether variation in immune regulation precedes disease development because they use standardized and well-documented exposures and measures. Increased insights into determinants of such a variation may provide further clues into the disease-causing chain of events in MS and other immunological diseases.

## Ethics approval

This study was approved by the regional ethics committee (REK South East, ref. no. 2016/1731) with informed consent for persons included from the national tuberculosis screening programme waived in the current study. Written informed consent for use of the information in the research and for data linkage was obtained during enrolment from all participants in the MS registry.

## Data availability

Pseudonymized data are not sharable according to Norwegian law. Further information about the data set is available from the corresponding author on reasonable request.

## Supplementary data

Supplementary data are available at *IJE* online.

## Author contributions

O.N. (guarantor) collected data, performed statistical analyses, interpreted data and drafted the manuscript. T.H. drafted the Introduction, interpreted data and revised the manuscript. H.S. designed the analytical strategy and revised the manuscript. K.M.M. interpreted data and revised the manuscript. E.H. interpreted data and revised the manuscript. J.D. interpreted data and revised the manuscript. H.M. designed the study, interpreted data and revised the manuscript.

## Funding

None.

## Conflict of interest

K.M.M. has received unrestricted research grants to his institution, scientific advisory board or speaker honoraria from Biogen, Sanofi, Merck, Novartis or Roche and has participated in clinical trials organized by Biogen, Sanofi, Merck, Novartis and Roche. T.H. has received speaker honoraria, research support/grants and participated in clinical trials for Biogen, Merck, Sanofi and Novartis, is a member of the scientific board of the Norwegian MS society and has received financial support from the Research Council of Norway (grant #250864). All other authors have none declared.

## Supplementary Material

dyac039_Supplementary_Data
