# Supplementary figures and images for: Strong tuberculin response after BCG vaccination is associated with low multiple sclerosis risk: a population-based cohort study
Source: Int J Epidemiol. 2022 Mar 12;51(5):1637–44. doi: 10.1093/ije/dyac039 (PMC9557857; doi:10.1093/ije/dyac039)

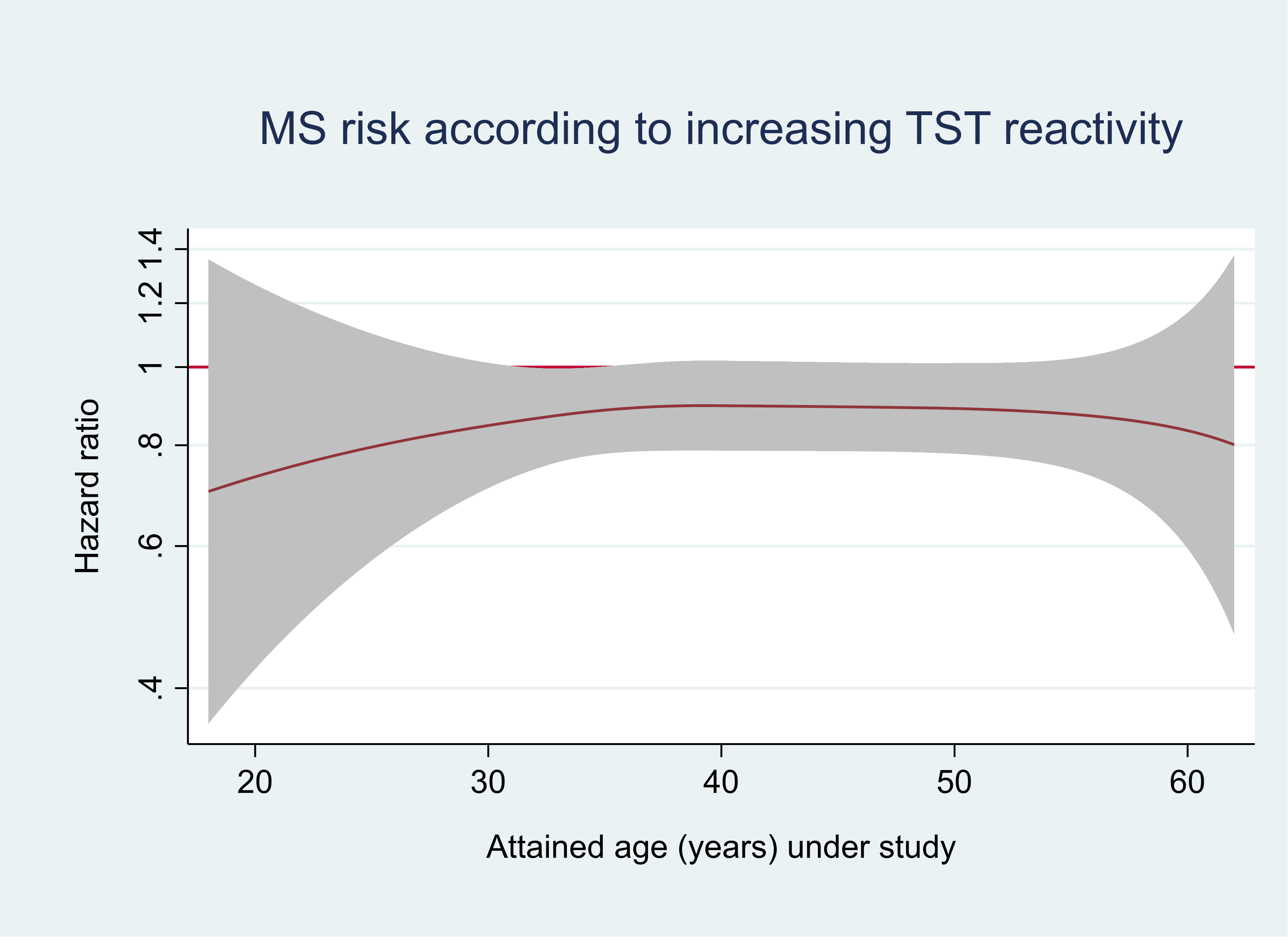

Supplement: dyac039_Supplementary_Data [file dyac039_supplementary_data.zip › ije-2021-10-1448-File007.tif]
